# Nighttime Cholecystectomies are Safe When Controlled for Individual Patient Risk Factors–A Nationwide Case–Control Analysis

**DOI:** 10.1007/s00268-021-06021-7

**Published:** 2021-03-18

**Authors:** Kian Merati-Kashani, Claudio Canal, Dominique Lisa Birrer, Pierre-Alain Clavien, Valentin Neuhaus, Matthias Turina

**Affiliations:** 1 Department of Surgery, Hospital of Maennedorf, Asylstrasse 10, CH-8708 Maennedorf, Switzerland; 2 Division of Trauma Surgery, Department of Traumatology, University Hospital Zurich, University of Zurich, Raemistrasse 100, CH-8091 Zurich, Switzerland; 3 Department of General and Transplant Surgery, University Hospital Zurich, University of Zurich, Raemistrasse 100, CH-8091 Zurich, Switzerland

## Abstract

**Background:**

The aim of this study was to evaluate if the time of day a cholecystectomy was performed affects in-hospital complication rates and mortality.

**Methods:**

A national quality measurement database was retrospectively studied. Study period was 2010 to 2017. The inclusion criteria were operatively treated cholecystitis or another benign disease of the gallbladder. Further, the time of day the operation was performed must have been documented. We defined nighttime as all interventions performed between 7PM until 6AM. A total of 11′459 patients were included. Development of any complication during hospitalization and in-hospital mortality was the main outcomes. The first part of the study was solely descriptive. In the second part, we applied a 1:1 case–control-matching. A matched group of 274 pairs were further investigated.

**Results:**

Only 8.4% of the procedures were performed during nighttime. Complications occurred in 6.7% of all patients. We found twice as many complications in the nighttime group compared to the daytime group. Mortality was 0.56% during daytime and 0.52% during nighttime. In a matched-pair analysis, however, we found no significant differences in the overall mortality rate nor in the occurrence of complications when comparing day- vs. nighttime operations.

**Conclusions:**

We found twice as many complications in the nighttime group (12%) compared to the daytime group (6.1%), mainly related to patient risk factors. In contrast to common apprehension, however, nighttime cholecystectomies were not associated with higher mortality rates.

**Supplementary Information:**

The online version contains supplementary material available at 10.1007/s00268-021-06021-7.

## Introduction

The standard treatment for symptomatic cholecystolithiasis as well as for acute cholecystitis is surgical removal of the gallbladder [1–3]. Today, up to 90% of all cholecystectomies are completed laparoscopically. The laparoscopic technique is associated with a faster recovery, a shorter length of stay and less complications such as pneumonia, pulmonary embolism, thrombosis and incisional hernias.

While the advantages of laparoscopy are well studied, controversies remain regarding the timing of the cholecystectomy. Early and same admission cholecystectomy for acute cholecystitis seems to be preferred due to lower morbidity rates, lower conversion rates, lower length of stay and lower hospital costs, even in cases with mild biliary pancreatitis [4–13]. An early operation can reduce the risk of recurrence or progression of the disease.

However, whether cholecystectomies should be performed during nighttime in patients with acute indications remains a controversial issue. X. Wu et al. compared nighttime vs. daytime cholecystectomies for acute cholecystitis [14]. They analyzed 1140 patient and showed an increased conversion rate to open surgery for nighttime cases, however, with no influence on the length of stay or complication rates. They concluded that cholecystectomies should be performed during daytime. This conclusion, however, is not universally accepted—S. Siada et al. in contrast showed that there is no higher risk of complications for laparoscopic cholecystectomies performed during nighttime hours [15].

The aim of this study was to determine whether the timing of surgery has any impact on in-hospital complication rates or mortality in cholecystectomy in a national cohort study.

## Material and methods

The prospective database of the Swiss working group for quality assurance in surgery (Arbeitsgemeinschaft für Qualitätssicherung in der Chirurgie “AQC” [16]) was queried to identify patients with an acute disease of the gallbladder requiring an operation. In Switzerland, over 70 hospitals provide standardized data of in-hospital surgical patients to the AQC. The AQC-database contains currently more than 1.5 million cases. To enter data for the AQC-database two forms must be filled in online with a) information on the inpatient treatment and b) on the operation(s). The recorded and for analysis available data are presented in Table 1 and 2. The World Health Organization's *International Statistical Classification of Diseases and Related Health Problems* (ICD-10) is used to code the diagnosis [17] and the Swiss operation classification “CHOP” for the procedures [18]. The study has been approved by the institutional review board–no special approval was needed due to de-identified data.

**Table 1:** Patient characteristics, day- vs. nighttime

Parameter		Total (*n* = 11,459)		Group daytime (*n* = 10,495)		Group nighttime (*n* = 964)		*p* value
		*n*	%	*n*	%	*n*	%	
Age (years)	mean ± SD	55 ± 17		55 ± 17		57 ± 18		0.013
Gender	male	4491	39	4074	39	417	43	0.007
	female	6968	61	6421	61	547	57	
ASA	I (healthy person)	3564	31	3311	32	253	26	< 0.001
	II (mild systemic disease)	6496	57	5938	57	558	58	
	III (severe systemic disease)	1351	12	1206	11	145	15	
	IV (severe systemic disease that is a constant threat to life)	46	0.40	39	0.37	7	0.73	
	V (moribund person who is not expected to survive without the operation)	2	0.017	1	0.0095	1	0.10	
Admission type	emergency	4382	38	3564	34	818	85	< 0.001
	registered, planned	7077	62	6931	66	146	15	
Insurance	statutory	9114	80	8301	79	813	84	< 0.001
	private	2345	21	2194	21	151	16	
Length of stay (days)	mean ± SD	4.0 ± 4.5		3.9 ± 4.2		5.3 ± 6.0		< 0.001
Length of stay preoperative (days)	mean ± SD	1.0 ± 2.1		1.0 ± 2.1		1.0 ± 2.0		n.s
Length of stay postoperative (days)	mean ± SD	3.0 ± 3.6		2.9 ± 3.4		4.3 ± 5.1		< 0.001
Duration ICU (hours)	mean ± SD	1.3 ± 16		1.2 ± 17		2.5 ± 14		< 0.001
Comorbidity	yes	3161	28	2837	27	324	34	< 0.001
Intubation	yes	808	7.1	758	7.2	50	5.2	0.018
Discharge	deceased	64	0.56	59	0.56	5	0.52	< 0.001
	at home	11,062	97	10,158	97	904	94	
	nursing home	74	0.65	60	0.57	14	1.5	
	old people's home	51	0.45	44	0.42	7	0.73	
	rehabilitation clinic	58	0.51	43	0.41	15	1.6	
	other	150	1.3	131	1.2	19	2.0	
Diagnosis	K80 Calculus of gallbladder with acute cholecystitis	2857	25	2382	23	475	49	< 0.001
	K80.1 Calculus of gallbladder with other cholecystitis	3826	33	3607	34	219	23	
	K80.2 Calculus of gallbladder without cholecystitis	2768	24	2706	26	62	6.4	
	K80.3 Calculus of bile duct with cholangitis	45	0.39	43	0.41	2	0.21	
	K80.4 Calculus of bile duct with cholecystitis	185	1.6	177	1.7	8	0.83	
	K80.5 Calculus of bile duct without cholangitis or cholecystitis	181	1.6	173	1.6	8	0.83	
	K80.8 Other cholelithiasis	148	1.3	147	1.4	1	0.10	
	K81 Cholecystitis	1304	11	1126	11	178	18	
	K82 Other diseases of gallbladder	121	1.1	110	1.0	11	1.1	
	K83 Other diseases of biliary tract	24	0.21	24	0.23	0	0	

SD: standard deviation; *ASA*, American Society of Anesthesiologists classification system; *n*.*s*., not significant

**Table 2:** Procedure characteristics, day- vs. nighttime

Parameter		Total (*n* = 11,459)		Group daytime (*n* = 10,495)		Group nighttime (*n* = 964)		*p* value
		*n*	%	*n*	%	*n*	%	
Surgeon class	senior attending	4171	36	3950	38	221	23	< 0.001
	junior attending	4413	39	3914	37	499	52	
	resident	2875	25	2631	25	244	25	
Type of surgery	laparoscopically	9715	85	9100	87	615	64	< 0.001
	conversion	1403	12	1112	11	291	30	
	open	341	3.0	283	2.7	58	6.0	
Duration surgery (minutes)	mean ± SD	86 ± 49		85 ± 48		99 ± 55		< 0.001
Complications	yes	763	6.7	645	6.1	118	12	< 0.001
Teaching	yes	2684	23	2466	23	218	23	0.021
Thromboembolism prophylaxis	thromboembolism prophylaxis	10,480	91	9575	91	905	94	0.017
	no thromboembolism prophylaxis	648	5.7	607	5.8	41	4.3	
	anticoagulation	331	2.9	313	3.0	18	1.9	
Antibiotics	no antibiotics	1089	9.5	1041	9.9	48	5.0	< 0.001
	prophylactic antibiotics (before start of surgery)	8376	73	7773	74	603	63	
	prophylactic antibiotics (after start of surgery)	150	1.3	133	1.3	17	1.8	
	antibiotic therapy	1844	16	1548	15	296	31	

*SD,* standard deviation

The inclusion criteria were operatively treated cholecystitis or another benign disease of the gallbladder, notably symptomatic cholecystolithiasis (ICD-diagnostic codes K80 to K83). The time of day a cholecystectomy was performed had to be recorded. (The start time of an operation is an optional field in the AQC-questionnaire.) The time span was 01/01/2010—12/31/2017. Exclusion criteria were missing data. A total of 11′459 patients were eventually included in our analysis.

The patients were stratified into two groups depending on the start of the surgical intervention: daytime (7AM until 6PM) and nighttime (7PM until 6AM).

Development of any complication during hospitalization and in-hospital mortality was the main outcomes.

To evaluate the influence of the daytime an operation was performed, we applied a case–control-matching. The goal was to match on confounding variables who account for pre-existing differences, to reduce selection bias and to improve internal validity. Using the case–control-matching feasibility in SPSS, we performed a one-to-one matching of our two different time groups (day- versus nighttime) sequentially on the basis of exact age, gender, ASA-Status (ASA I-V, however, for ASA IV and V we had no matches), admission type, insurance status, the presence of a comorbidity, the exact diagnosis, the training level of the surgeon and the type of surgery. A matched group of 274 pairs fulfilled the matching. (Table 3).

**Table 3:** Characteristics of matched daytime with nighttime cases

Parameter		Group daytime (*n* = 274)		Group nighttime (*n* = 274)		*p* value
		*n*	%	*n*	%	
Age (years)	mean ± SD	55 ± 16		55 ± 16		1.0
Gender	male	110	40	110	40	1.0
	female	164	60	164	60	
ASA	I (healthy person)	52	19	52	19	1.0
	II (mild systemic disease)	198	72	198	72	
	III (severe systemic disease)	24	8.8	24	8.8	
Admission type	emergency	215	78	215	78	1.0
	registered, planned	59	22	59	22	
Insurance	statutory	251	92	251	92	1.0
	private	23	8.4	23	8.4	
Comorbidity	yes	65	24	65	24	1.0
Diagnosis	K80 Calculus of gallbladder with acute cholecystitis	160	58	160	58	1.0
	K80.1 Calculus of gallbladder with other cholecystitis	63	23	63	23	
	K80.2 Calculus of gallbladder without cholecystitis	14	5.1	14	5.1	
	K81 Cholecystitis	37	14	37	14	
Surgeon class	senior consultant, attending surgeon	53	19	53	19	1.0
	junior consultant	160	58	160	58	
	resident	61	22	61	22	
Type of surgery	laparoscopically	251	92	251	92	1.0
	conversion	23	8.4	23	8.4	

*SD*, standard deviation; *ASA*, American Society of Anesthesiologists classification system

The data were downloaded via an online tool (AdjumedAnalyze, Adjumed Services AG, Zurich, Switzerland) and analyzed by using the Statistical Package for Social Sciences (SPSS, Version 24, IBM Corp., Armonk, New York, the USA).

The first part of the study was descriptive (continuous and categorical data). The second part was bivariate analysis. The normality of the data was assessed with the Kolmogorov–Smirnov test. The Chi-square, Mann–Whitney U and Fisher tests, where applicable, were used to do bivariate analysis. McNemar tests (for dichotomous categorical variables) and paired t-tests (for continuous variables) were used in bivariate analysis comparing our two matched groups (Table 4).

**Table 4:** Outcome details of matched daytime with nighttime cases

Parameter		Group daytime (*n* = 274)		Group nighttime (*n* = 274)		*p* value
		*n*	%	*n*	%	
Length of stay preoperative (days)	mean ± SD	1.4 ± 1.9		0.69 ± 1.2		< 0.001
Length of stay postoperative (days)	mean ± SD	3.0 ± 2.1		3.9 ± 4.0		< 0.001
Duration ICU (hours)	mean ± SD	1.0 ± 7.2		2.6 ± 15		0.128
Intubation	yes	26	9.5	16	5.8	0.108
Discharge	deceased	2	0.73	2	0.73	0.906
	at home	261	95	260	95	
	nursing home	2	0.73	3	1.1	
	old people's home	3	1.1	2	0.73	
	rehabilitation clinic	1	0.36	3	1.1	
	other	5	1.8	4	1.5	
Duration surgery (minutes)	mean ± SD	97 ± 49		92 ± 42		0.164
Complications	yes	23	8.4	27	9.9	0.527
Teaching	yes	68	25	50	18	0.011

*SD*, standard deviation

Factors associated with mortality were assessed in bivariate analysis only because of the low number of deaths. Risk factors for complications were evaluated in a stepwise backward likelihood logistic regression analysis. Significant (*p* < 0.05) or nearly significant factors (*p* < 0.1) in bivariate analysis were chosen as potential confounders.

A post hoc power analysis for complications determined that the total sample size of 548 patients provided 81% power.

## Results

### The study population

A total of 11′459 patients were examined in this study. The mean age was 55 ± 17 years. Sixty-one percent of the patients were female. Thirty-eight percent of the patients were admitted as emergencies. Fifty-seven percent of the examined patients had an ASA score of II (mild systemic disease). Twenty-eight percent of all patients suffered from at least one comorbidity. The most frequent diagnosis was K80.1 (calculus of the gallbladder with cholecystitis and with or without bile duct obstruction). Most operations were performed by junior attendings (39% of all cases), followed by senior attendings (36%). Eighty-five percent of the cholecystectomies were performed laparoscopically.

Complications arised in 6.7 percent of all patients. The most common complications were urinary retention, pneumonia, pancreatitis, cardiac arrhythmia, respiratory failure, sepsis and urinary tract infection. The most common intraoperative complications reported were lesion to the liver or liver bed, a gastrointestinal lesion and a lesion to the bile duct. The most common postoperative complications reported were wound healing disorder, post-bleeding anemia and bile fistula. The complication rates were similar between the two groups. (Supplementary Table 1).

Sixty-four patients (0.56%) died during the hospitalization. The average length of stay was 4.0 ± 4.5 days. (Tables 1 and 2).

### Timing of surgery and outcome

Only 8.4% of the procedures were performed during nighttime. The patients in the nighttime group had significantly more comorbidities, a slightly higher ASA-score, were more than twice as often assigned as emergency and were more often covered by statutory insurance plans than the patients operated on during daytime. (Table 1) Junior attendings and residents carried out 77% of all operations during nighttime. Duration of surgery was significantly longer in the nighttime group compared to the daytime group and lasted on average 14 min longer. (Table 2).

We found twice as many complications in the nighttime group (12%) compared to the daytime group (6.1%), which was significant. In-hospital mortality was 0.56% (*n* = 59) during daytime and 0.52% (*n* = 5) during nighttime.

### Matched-pair analysis

The matching produced two groups with no significant differences in length of stay, time in the ICU, the need for intubation and duration of surgery. In our matched group, we found more teaching interventions in the daytime group than in the nighttime group.

The time of surgery (day- vs. nighttime) had no influence on the primary outcome: The complication rates were similar in our matched-pair analysis. In addition, the overall mortality rate in our matched-pairs analysis was 0.73% (0.73% in the daytime group and 0.73% in the nighttime group, n.s.). (Table 4).

### Bivariate and multivariate analysis

Mortality was associated with higher age, higher ASA scores, longer ICU and hospital stays. Patients who died suffered more complications, underwent rather open than laparoscopic surgery and received more often antibiotic therapy (vs. prophylactic only) than patients who survived.

A higher age, a higher ASA-score, open surgery and conversion vs. laparoscopic operation, longer duration of the cholecystectomy, operation at nighttime vs. daytime, antibiotic therapy vs. no antibiotics and a private insurance status vs. statutory, were significant predictors of general and postoperative complications in multivariate analysis (*R* square = 0.22) (Table 5 and 6).

**Table 5:** Predictors for complications in all patients

Parameter	Sig	OR	95% C.I.for EXP(B)	
			Lower	Upper
ASA IV (vs. ASA I)	< 0.001	5.427	2.190	13
ASA III (vs. ASA I)	< 0.001	1.878	1.381	2.555
Open (vs. laparoscopically)	0.007	1.622	1.138	2.312
Antibiotic therapy (vs. no antibiotics)	0.021	1.607	1.074	2.406
Conversion (vs. laparoscopically)	0.004	1.403	1.116	1.764
Nighttime (vs. daytime)	0.024	1.346	1.039	1.743
Insurance status private (vs. statutory)	0.011	1.338	1.070	1.672
ASA II (vs. ASA I)	0.021	1.330	1.044	1.693
Anticoagulation (vs. thromboembolism prophylaxis)	0.194	1.308	0.872	1.960
Age (years)	< 0.001	1.015	1.009	1.022
Duration surgery (minutes)	< 0.001	1.014	1.013	1.016
Antibiotic prophylaxis (before start of surgery) (vs. no antibiotics)	0.858	0.925	0.394	2.171
Antibiotic prophylaxis (after start of surgery) (vs. no antibiotics)	0.334	0.828	0.565	1.214
No thromboembolism prophylaxis (vs. thromboembolism prophylaxis)	< 0.001	0.217	0.096	0.492

Added in the analyze: age, duration of surgery, gender, ASA score (without ASA V), admission type, insurance status, comorbidity, diagnosis, surgeon class, type of surgery, teaching, thromboembolism prophylaxis, antibiotic therapy and day- or nighttime

*OR*, Odds ratio

**Table 6:** Factors associated with mortality (bivariate analysis) in all patients

Parameter		Survivors (n = 11,395)		Non-survivors (n = 64)		*p* value
		*n*	%	*n*	%	
Age (years)	mean ± SD	55 ± 17		63 ± 19		< 0.001
ASA	I (healthy person)	3542	31	22	34	< 0.001
	II (mild systemic disease)	6472	57	24	38	
	III (severe systemic disease)	1338	12	13	20	
	IV (severe systemic disease that is a constant threat to life)	41	0.36	5	7.8	
	V (moribund person who is not expected to survive without the operation)	2	0.018	0	0	
Length of stay (days)	mean ± SD	4.0 ± 4.4		14 ± 16		< 0.001
Duration ICU (hours)	mean ± SD	1.0 ± 12		48 ± 145		< 0.001
Diagnosis	K80 Calculus of gallbladder with acute cholecystitis	2834	25	23	36	0.004
	K80.1 Calculus of gallbladder with other cholecystitis	3813	33	13	20	
	K80.2 Calculus of gallbladder without cholecystitis	2756	24	12	19	
	K80.3 Calculus of bile duct with cholangitis	45	0.39	0	0	
	K80.4 Calculus of bile duct with cholecystitis	180	1.6	5	7.8	
	K80.5 Calculus of bile duct without cholangitis or cholecystitis	179	1.6	2	3.1	
	K80.8 Other cholelithiasis	145	1.3	3	4.7	
	K81 Cholecystitis	1299	11	5	7.8	
	K82 Other diseases of gallbladder	121	1.1	0	0	
	K83 Other diseases of biliary tract	23	0.20	1	1.6	
Type of surgery	laparoscopically	9664	85	51	80	0.001
	conversion	1397	12	6	9.4	
	open	334	2.9	7	11	
Complications	yes	739	6.5	24	38	< 0.001
Antibiotics	no antibiotics	1082	9.5	7	11	< 0.001
	prophylactic antibiotics (before start of surgery)	8348	73	28	44	
	prophylactic antibiotics (after start of surgery)	149	1.3	1	1.6	
	antibiotic therapy	1816	16	28	44	

*SD*, standard deviation; *ASA*, American Society of Anesthesiologists classification system; n.s., not significant

## Discussion

Most cholecystectomies are completed in a planned and elective setting during daytime. Obviously, some patients were unable to receive their elective cholecystectomy during the elective daytime schedule, and their operation was then performed by the on-call staff during nighttime so this patient would not have to be discharged and rescheduled for a later date. Under certain circumstances (symptomatic cholelithiasis or acute/gangrenous cholecystitis), it is necessary to perform the surgery also off-hours, in our study defined as the time between 7 PM until 6 AM to reduce the risk of recurrent complications. The aim of this study was to determine whether the timing of surgery has any impact on in-hospital outcome.

In summary, our data showed equal mortality but a higher morbidity in patients operated at night versus patients operated during daytime. However, patients who had undergone surgery in the off-hours were significantly older, had a higher ASA score, had more comorbidities and were more frequently operated on by junior attendings. And these patients had twice as many complications. Yet, a matched-pair analysis controlling for several risk factors such as patient age, gender, ASA class, severity of the disease and type of surgery found that outcomes did indeed not differ between day- and nighttime cholecystectomies. Therefore, we conclude that patient-related factors play a role in the observed increase in postoperative morbidity, but not the time of surgery. Alternatively, factors not accounted for in our administrative database, such as surgeon fatigue, may influence results of operations in patients of otherwise similar characteristics. This is in accordance with a study from the Netherlands. Geraedts et al. posed the question whether an off-hour laparoscopic cholecystectomy would really increase postoperative morbidity [19]. In her study, data from 1553 laparoscopic cholecystectomies between 2014 and 2016 were prospectively collected and analyzed. Similar to our study, the number of nighttime procedures was 9.3%, compared to 8.4% in our case. The key message of her study was that a nighttime laparoscopic cholecystectomy is not an independent risk factor for increased postoperative morbidity, and therefore, the time of surgery is only a relative contraindication. However, Blohm et al. found somewhat higher frequencies of adverse events in patients operated on admission day and emphasized the importance of optimizing the patient before surgery [20]. Wu et al. showed that nighttime cholecystectomy is associated with an increased conversion to open surgery without decrease in length of stay or complications [14]. This is consistent with our findings. We found a considerably higher conversion rate in the nighttime group (30 vs. 11%). The reasons seem multifactorial, the experience of the surgeon may contribute to this finding. However, we also think that this shows good surgical judgment and does not always represent a complication.

These statements are further strengthened by our multivariate and bivariate analysis.

Even in a subgroup analysis with a narrower definition of nighttime (11 PM to 6 AM), we found a similar cohort concerning age, sex and ASA with the same complication rate and from these 331 patients operated during nighttime none died during their stay in-hospital. (Supplementary Table 2 and 3).

The limitations of our large study include among others a selection bias in that we cannot deduce the individual decisions that have led to a nighttime cholecystectomy as opposed to a scheduled operation. Presumably, the reasons are in the context of the acute emergency and the vital threat.

## Conclusion

We found twice as many complications in the nighttime group (12.2%) compared to the daytime group (6.1%), which was mainly based on patients being sicker and not on time of surgery. There is no significant effect of the time of day a cholecystectomy is performed regarding mortality. In contrast to common apprehension, however, nighttime cholecystectomies were not associated with higher mortality rates in our national sample. Urgent operations may therefore be performed at night without compromise in patient safety by surgeons trained and experienced in technically difficult cholecystectomies.

## Supplementary Information

Below is the link to the electronic supplementary material.

Supplementary file1 (XLSX 14 KB)

Supplementary file2 (XLSX 15 KB)

Supplementary file3 (XLSX 13 KB)
